# Diurnal-and sex-related difference of metallothionein expression in mice

**DOI:** 10.1186/1740-3391-10-5

**Published:** 2012-07-24

**Authors:** Dan Zhang, Tao Jin, Yi-qiao Xu, Yuan-Fu Lu, Qin Wu, Yu-Kun Jennifer Zhang, Jie Liu

**Affiliations:** 1 Dept of Pharmacology and Key Lab of Basic Pharmacology of Guizhou, Zunyi Medical College, Zunyi, Guizhou 563003, China; 2 University of Kansas Medical Center, Kansas City, KS 66160, USA

**Keywords:** Metallothionein, Diurnal variation, Mouse liver, Kidney, Blood

## Abstract

**Background:**

Metallothionein (MT) is a small, cysteine-rich, metal-binding protein that plays an important role in protecting against toxicity of heavy metal and chemicals. This study was aimed to define diurnal and sex variation of MT in mice.

**Methods:**

Adult mice were maintained in light- and temperature-controlled facilities for 2 weeks with light on at 8:00 and light off at 20:00. The blood, liver, and kidneys were collected every 4 h during the 24 h period. Total RNA was isolated, purified, and subjected to real-time RT-PCR analysis and MT protein was determined by western blot and the Cd/hemoglobin assay.

**Results:**

The diurnal variations in mRNA levels of MT-1 and MT-2in liver were dramatic, up to a 40-foldpeak/trough ratio. MT mRNA levels in kidneys and blood also showed diurnal variation, up to 5-fold peak/trough ratio. The diurnal variation of MT mRNAs resembled the clock gene albumin site D-binding protein (Dbp), and was anti-phase to the clock gene Brain and Muscle ARNT-like Protein 1 (Bmal1) in liver and kidneys. The peaks of MT mRNA levels were higher in females than in males. Hepatic MT protein followed a similar pattern, with about a 3-fold difference.

**Conclusion:**

MT mRNA levels and protein showed diurnal- and sex-variation in liver, kidney, and blood of mice, which could impact the body defense against toxic stimuli.

## Background

Metallothionein (MT) was discovered in 1957 from equine kidney, and was characterized by its metal-binding capacity and high sulfhydryl content [1]. MT has four isoforms termed MT-1, MT-2, MT-3 and MT-4 and is ubiquitous in mammals [2]. MT has been shown to have multiple functions such as in the homeostasis of tissue copper and zinc, in the detoxification of heavy metals, in the modulation of immune function, and in the scavenge of free radicals [3].

When cadmium was administered during the dark phase of the daily cycle, the kidneys and liver contained more cadmium and higher levels of MT [4]. It has been shown that circadian clock-controlled genes in *Neurosporacrassa* are late-night to early morning specific. One of them, *ccg-12*, encodes copper metallothionein [5]. Two reports indicate that cadmium-induced mortality showed a circadian variation in mice [6,7]. The circadian variation to cadmium toxicity could be associated with diurnal variation of metallothionein. However, no information on diurnal variation of MT in mammals is available. This study was initiated to fill that gap.

We have recently demonstrated the circadian variations in hepatic antioxidant components including the Nrf2 pathways, glutathione systems, and antioxidant enzymes in male and female mice [8], thus, it would be of interest to examine whether the diurnal variations exist for the basal levels of MT mRNA. We firstly verified the circadian rhythms of 3 clock genes, followed by examination of diurnal variations of the major MT isoforms (MT-1 and MT-2) in major organs (liver, kidney, and blood) of Kunming mice. The results revealed for the first-time a dramatic diurnal variation of the MT mRNA, which could have important pharmacological and toxicological significance.

## Materials and Methods

### Animals

Male and female adult Kunming (KM) mice [9] (n = 24/sex), weighing 18–22 g, were obtained from the Animal Breeding Center of the Third Military Medical University (Scsk2007-0001, Chongqing, China), and maintained in the SPF-grade (equivalent to AAALAC accreditation) animal facilities at Zunyi Medical College. All animal care and experimental protocols complied with the Animal Management Guidelines of the Chinese Ministry of Health. Mice were acclimated in a temperature-humidity controlled facility with a standard 12-h light/dark schedule (lights on at 8:00 and off at 20:00). All animals had free access to rodent chow and drinking water. After acclimatizing all animals for 2 weeks, four animals per sex were sacrificed at six time points (2:00, 6:00, 10:00, 14:00, 18:00, and 22:00). Blood, liver, and kidneys were collected. Approximately 0.5 ml blood was mixed with 0.5 ml TRIzol. Livers and kidneys were frozen in liquid nitrogen and stored at −80°C prior to analysis.

### RNA Isolation and Real-time RT-PCR analysis

Approximately 50–100 mg of liver and kidney tissues were homogenized in 1 ml TRIzol (TakaRa Biotechnology, Dalian, China). Total RNA was extracted according to the manufacturer’s instructions, followed by purification with a Total RNA(Mini) Kit (Watson Biotechonologins, Shanghai, China). The quality and quantity of RNA were determined by the 260/280 ratio and gel-electrophoresis. Purified RNA was reverse transcribed with the High Capacity Reverse Transcriptase Kit (Applied Biosystems, Foster City, CA, USA). The primers were designed with Primer3 software and listed in Table 1. The Power SYBR Green Master Mix (Applied Biosystems, Foster City, CA, USA) was used for real-time RT-PCR analysis. The expression of genes was first normalized with β-actin of the same sample, and the relative transcript levels were calculated as percentage of β-actin.

**Table 1:** Primer sequence for real-time RT-PCR analysis

**Gene**	**Genbank**	**Forward**	**Reverse**
β-actin	V01217	TGACCGAGCGTGGCTACAG	GGGCAACATAGCACAGCTTCT
Bmal1	NM_007489	ACGACATAGGACACCTCGCAGA	CGGGTTCATGAAACTGAACCATC
Cry1	NM_007771	GGATCCACCATTTAGCCAGACAC	CATTTATGCTCCAATCTGCATCAAG
Dbp	NM_016974	CTGGCCCGAGTCTTTTTGC	CCAGGTCCACGTATTCCACG
MT-1	NM_013602	CTCCGTAGCTCCAGCTTCAC	AGGAGCAGCAGCTCTTCTTG
MT-2	NM_008630	CCGATCTCTCGTCGATCTTC	AGGAGCAGCAGCTTTTCTTG

### Hepatic MT protein determination

MT protein concentrations in livers were determined by the cadmium–hemoglobin assay. Liver tissues were homogenized in physiological saline (1:10, wt: vol), followed by centrifugation at 10,000 × g for 10 min. Aliquots of the supernatant (0.1 ml) were mixed with CdCl_2_ solution (2 μg Cd/ml;100 μl),followed by the addition of 50 μl of 2% hemoglobin. The mixture was heated in boiling water for 90 seconds and centrifuged (12,000 g, 5 min). Then another 50 μl of 2% hemoglobin was added, boiled and centrifuged again. The supernatant (100 μl) was taken for determination of Cd by Atomic Absorption Spectrometry (Varian, Montreal, Canada).

### Western blot analysis

The frozen tissue samples of the same group were pooled and put into 1 ml lysing buffer, which contain RIPA, PMSF(Beyotime, Shanghai, China), and protease inhibitor cocktail (Calbiochem, San Diego, USA). The homogenates were centrifuged (10000 g, 10 min) and the supernatants collected. Protein concentrations were determined using the Enhanced BCA Protein Assay Kit (Beyotime, Shanghai, China). The samples were boiled with Sample Loading Buffer for 10 min (Beyotime, Shanghai, China). Samples (100 μg) were separated on 15% SDS-PAGE gels and transferred to PVDF membranes. The membranes were blocked and incubated with anti-metallothionein antibody (Abcam, Cambridge, USA) at dilution1:1000 overnight at 4°C, followed by incubation with an appropriate horseradish peroxidase conjugated secondary antibody. The membranes were detected using a chemiluminescence kit (Thermo, USA). β-Actin was used to normalize protein loading.

### Statistical analysis

Special analysis of circadian rhythms has been documented and used for rhythm analysis worldwide [10]. In the present study, diurnal variation of MT was analyzed by the intergroup average cosine algorithm method with the help of Dr. Ding-Jun Cai (Chengdu Traditional Chinese Medical University, Statistics Department). Data were presented as mean and SEM. Sex-difference was analyzed by the one-way ANOVA, followed by Student^’^s *t*-test. P < 0.05 was considered statistically significant.

## Results

### Diurnal variations of clock gene Bmal1 and Dbp mRNA

**Figure 1:**
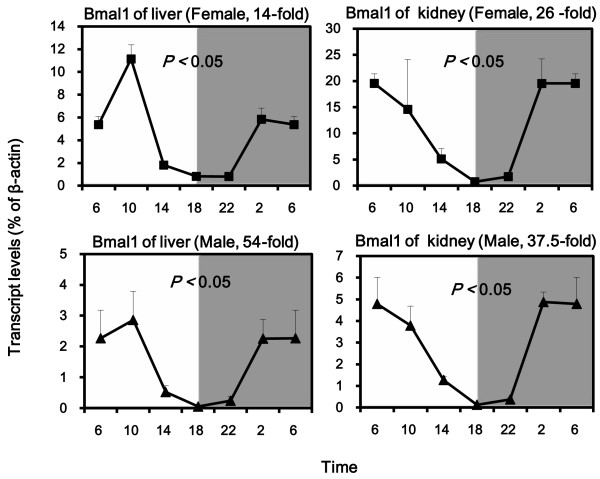
**Diurnal variations of mRNA levels of the clock gene Bmal1 in adult female and male KM mouse livers and kidneys (n = 4/sex/time point). **Values are means ± SEM. Circadian (t = 24 h) rhythms was confirmed by the cosine algorithm method (p < 0.05).

To verify the circadian clock gene expression pattern in KM mice, we analyzed the clock gene Bmal1 in livers and kidneys of KM mice using real-time quantitative RT-PCR. The expression of Bmal1 had a nadir at 18:00 and a peak at 6:00. The differences of the nadirs and peaks were 14-fold for females and 54-fold for males in liver; while in kidneys were 26-fold for females and 37.5-fold for males. Statistically significant 24 h rhythms were confirmed by cosine algorithm method (*p* < 0.05) (Figure 1).

**Figure 2:**
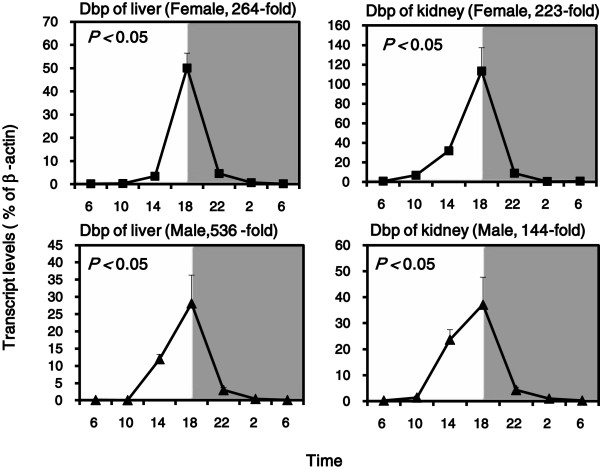
**Diurnal variations of mRNA levels of the clock Dbp in adult female and male KM mouse livers and kidneys (n = 4/sex/time point). **Values are means ± SEM. Statistically significant 24 h rhythms was confirmed by the cosine algorithm method (*p *< 0.05).

The expression of another clock gene Dbp was antiphase to Bmal1. Statistically significant 24 h rhythms were confirmed by the cosine algorithm method (*p* < 0.05) (Figure 2). The mRNA of Dbp was markedly increased at 14:00 and by 18:00 reached a peak. The peak was 264-fold higher than the nadir in the livers of female mice, and 536-fold in males. Dbp also expressed in kidney with the same pattern as in liver, with 223-fold in females and 114-fold in males.

### Diurnal variations of hepatic MT-1 mRNA in the liver and kidney

**Figure 3:**
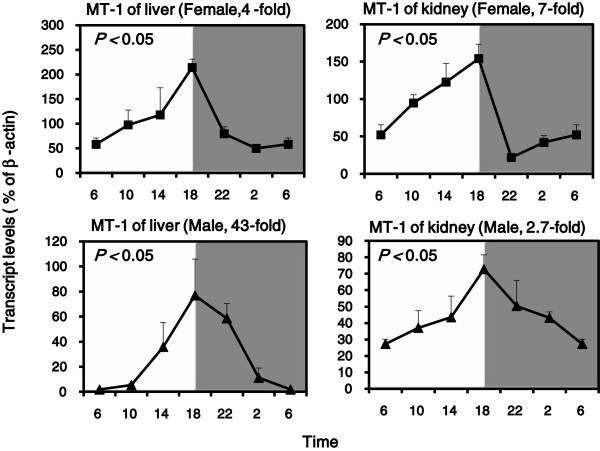
**Diurnal variations of mRNA levels of MT-1 in adult female and male KM mouse livers and kidneys (n = 4/sex/time point). **Values are means ± SEM. The rhythms was validated by the cosine algorithm method (*p *< 0.05).

The diurnal variation pattern of MT-1 mRNA resembles the clock gene Dbp. Statistically significant rhythms were confirmed by cosine algorithm method (*p* < 0.05) (Figure 3). The mRNA of MT-1 reached a peak at 18:00 and a nadir at 6:00. Females showed higher expression levels at 18:00 but males showed more dramatic circadian variations. The diurnal variation of MT-1 mRNA in the liver was 4-fold in females and 43-fold in males, while in kidney it was 7-fold in females and 2.7-fold in males.

### Diurnal variations of MT-2 mRNA in liver and kidney

**Figure 4:**
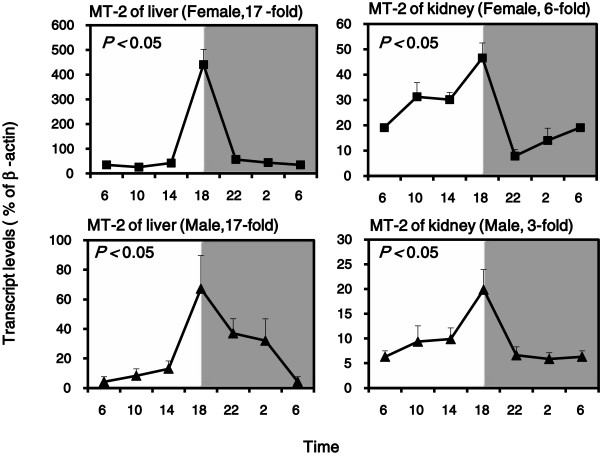
**Diurnal variations of mRNA levels of MT-2 in adult female and male KM mouse livers and kidneys (n = 4/sex/time point). **Values are means ± SEM. Significant circadian rhythm was confirmed by the cosine algorithm method (*p *< 0.05).

In mammals, MT-1 and MT-2 are coordinately regulated, and have similar functions [11]. The diurnal variation pattern of MT-2 mRNA expression resembles that of MT-1.The diurnal variations of MT-2 in the liver were 17-fold for females and 17-fold for males, while in the kidney were 6-fold for females and 3-fold for males. Statistically significant 24 h rhythms were confirmed by cosine algorithm method (*p* < 0.05) (Figure 4).

### Diurnal variations of MT-2 and Cry1 mRNA in blood

**Figure 5:**
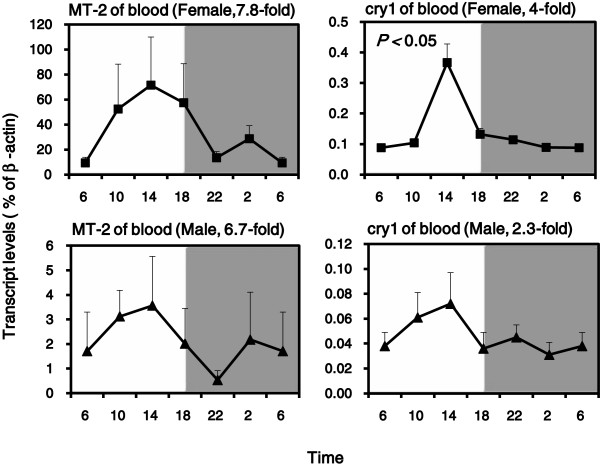
**Diurnal variations of MT-2 and Cry1 mRNA levels in adult female and male KM mouse blood (n = 4/sex/time point). **Values are means ± SEM. Significant circadian rhythm for Cry1 expression in females was confirmed by the cosine algorithm method (*p *< 0.05).

The circadian pattern of MT-2 mRNA in blood is slightly different from that of Dbp (data not shown), but resembled the clock gene cryptochrome 1(Cry1). Cry1 expression in females had a 24 h rhythms (4-fold) by cosine algorithm method (*p* < 0.05), but not for males (2.3-fold) (Figure 5). The nadir and peak differences for MT-2 mRNA in blood were 7.8-fold for females with 6.7-fold for males. Although no statistically significant rhythm for MT-2 mRNA in blood was detected by the cosine algorithm method due to individual variations, similar rhythm trend is clear.

### Diurnal variations of hepatic MT protein

**Figure 6:**
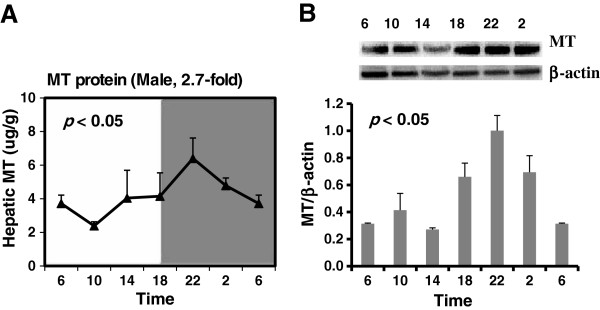
**Diurnal variations of hepatic MT protein in adult male KM mice. (A) **was determined by the Cd/hemoglobin assay(n = 4/sex/time point). **(B)** was determined by western blot (n = 4/sex/time point). Values are means ± SEM. Significant circadian rhythm was confirmed by the cosine algorithm method (*p *< 0.05).

MT protein was determined by western blot and the Cd/hemoglobin assay and the results were similar. Hepatic MT protein, with a peak at 22:00, showed about a 3-fold peak/trough ratio for males. MT protein reached a peak 4 h later after the peak of MT mRNA expression but to a lesser extent. The rhythms were validated by cosine algorithm method (*p* < 0.05) (Figure 6).

### Sex differences of clock and MT mRNA expression in liver, kidney and blood

**Figure 7:**
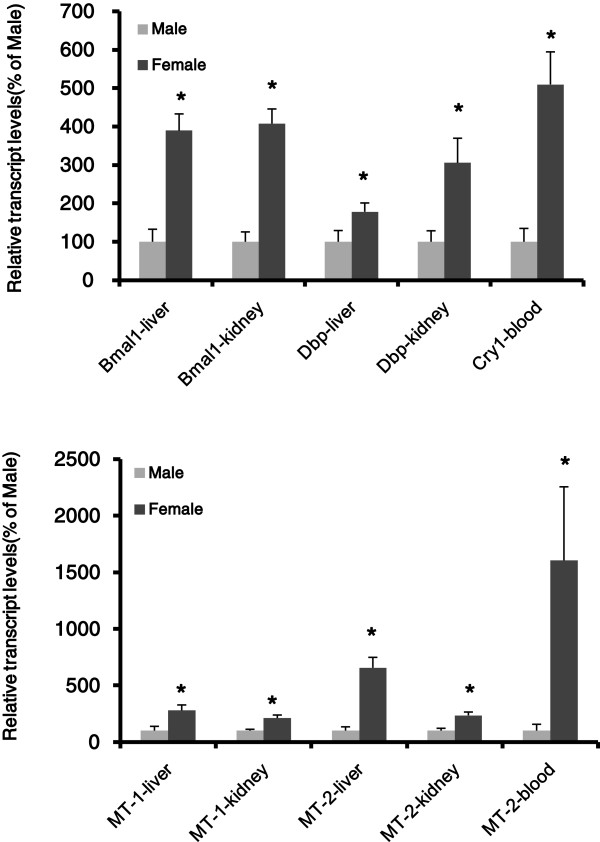
**Sex differences in mRNAs levels of clock and MT at their peak in males and females (n = 4/sex/time point). **Values are means ± SEM, and setting males as 100%. Significance was determined by Student’s t test (*p < 0.05 *vs *males).

The peak expressions of the clock and MT mRNA in liver, kidney and blood of male and female mice were compared and summarized in Figure 7. In general, females showed higher expression than males (Student’s *t* test, *p* < 0.05) (Figure 7).

## Discussion

The present study depicts the diurnal variations of MT during a 24 h period in liver (40-fold), kidney (7-fold), and blood (7-fold) of KM mice. The diurnal variations of MT genes resemble the clock genes Dbp and Cry1, but were antiphase to Bmal1. MT protein also showed diurnal variation, but to a lesser extent (3-fold) than mRNA. In general, female mice had higher MT mRNA levels than males. Similar findings were also observed in the inbred male C57BL/6 mouse liver (data not shown), fortifying this phenomenon.

Circadian rhythms in mammals are under control of a rhythm generator located in the hypothalamic suprachiasmatic nuclei (SCN) [12,13]. At the cellular level, circadian rhythms are driven by the transcription/translation-based negative feedback loops regulating the cyclic expression of clock genes (such as Cry1, Bmal1, Dbp, *etc.*) [14]. Our study verified he rhythms of the above 3 classic clock genes (Bmal1, Dbp and Cy1) in KM mice (Figures 1, 3, and 6), and the patterns of their expression were in agreement with the literature [15]. The clock gene expression pattern verifications validate the further examination of diurnal variations of metallothionein.

The liver is the major organ of drug metabolism and detoxification. MT acts as a part of the antioxidant defense mechanism against oxidative damage caused by toxic stimuli [3,16]. Mice deficient in MT were more susceptible than wild-type mice to hepatotoxicity of cadmium, CCl_4_, acetaminophen [16], arsenic [17], and rifampicin [18]. These data demonstrate that MT plays an important role in the defense against toxicity stimuli. In contrast, down-regulation of MT has been reported in hepatocellular carcinoma [19,20]. Thus, to define the circadian variations of MT is of toxicological and clinical significance. To our knowledge, this is the first report on circadian variation of MT in livers of mice.

It has long been known that kidney is a target organ of cadmium and long-term Cd exposure causes renal tubular dysfunction in occupational exposed and the general population [21]. Renal cadmium is detoxified by MT in the kidney [3,16], and the present study also showed circadian variation of MT in the kidney. This could impact not only Cd nephrotoxicity [22], but also other nephrotoxicants as well. MT has been implicated to play important roles in protecting against oxidative stress induced not only by cadmium [16], but also by organic chemicals such as adriamycin [23].

Cd-induced sensitivity to *Paramecium tetraurelia* also showed diurnal-variations [24]. Whether the circadian variations of organisms to cadmium are due to MT requires further investigation. The circadian variations of MT gene expression could influence the sensitivity of mammals to environmental chemicals, but also to oxidative stress.

The blood MT-mRNA could be used as a sensitive biomarker for exposure to cadmium [25], arsenic intoxication [26] and several other heavy metals [27]. The MT levels in blood can be considered as a promising biomarker for diagnosis, prognosis and therapeutic efficiency evaluation of childhood tumors [28]. The potential role of MT as a prostate cancer marker has been proposed [29,30]. This study depicts that mRNA levels of MT in blood also showed a diurnal variation, with a peak at 14:00,and peak/trough differences up to 7.8-fold. Thus it is important to realize for blood sample collection at designated times of the day to avoid circadian variations in MT gene expression when using MT mRNA as a biomarker.

In summary, these results demonstrate dramatic diurnal variations of MT-1 and MT-2gene expression in liver, kidney, and blood of KM mice. The diurnal variations in MT mRNA could be an important factor affecting the magnitude and progression in responses to toxic stimuli.

## Competing interests

The authors declare that they have no competing interests.

## Authors’ contributions

JL designed the research; DZ, TJ, YX, YL, and QW performed the research; DZ and YX analyzed the data; JL and DZ wrote the paper, JY Zhang helped generation of the idea and experimental design. All authors read and approved the final manuscript.
